# Differences Between Patients With Diabetes Mellitus and Those Without in Cases of Necrotizing Fasciitis

**DOI:** 10.7759/cureus.36576

**Published:** 2023-03-23

**Authors:** Daniel T DeGenova, Scott S Hyland, Tucker Peabody, Nolan P Schmitz, Devon Myers, Robert Patterson, Jignesh Patel

**Affiliations:** 1 Orthopedic Surgery, OhioHealth System, Columbus, USA; 2 Orthopedic Surgery, Naval Medical Center Portsmouth, Portsmouth, USA

**Keywords:** type ii diabetes, infections, diabetes, lrinec, necrotizing fasciitis

## Abstract

Purposes

This study aims to evaluate further differences between patients with diabetes and those without who have been diagnosed with necrotizing fasciitis (NF) to assist clinicians in improving morbidity and mortality.

Methods

All patients diagnosed with NF of an extremity were retrospectively reviewed and divided into two groups based on a diagnosis of diabetes. Patient charts were reviewed to obtain multiple variables, which were compared between groups.

Results

From 2015 to 2021, 115 patients underwent surgical intervention due to concern for NF of an extremity with 92 patients included for data computation. Patients with diabetes had an average Laboratory Risk Indicator for Necrotizing Fasciitis (LRINEC) score of 9.02 compared to 7.24 (p=0.02). Patients with diabetes had a significantly higher rate of undergoing amputation when diagnosed with NF (p<0.0001). The mortality rate for diabetes and non-diabetes cohorts were 30.9% and 18.9%, respectively (p=0.2).

Conclusion

This study demonstrated that patients with diabetes with confirmed NF of an extremity had a significantly higher LRINEC score were more likely to undergo an amputation primarily, and were more likely to have a polymicrobial infection compared to those without. The overall mortality rate of NF was 26.1%.

## Introduction

Necrotizing fasciitis (NF) is a rare soft tissue infection that spreads rapidly along fascial planes, often requiring emergent operative management [1-3]. There are approximately 500 to 1,000 cases of NF in the United States annually, with mortality estimated to range from 6% to 76% [3]. The extremities are more commonly involved than the trunk; however, the latter carries a higher mortality risk [4,5].

Despite the severity of the disease, the diagnosis of NF can be difficult with the diagnosis being missed in 85% to 100% of patients [6]. To assist in distinguishing NF from non-necrotizing soft tissue infections, a diagnostic scoring system has been proposed, the Laboratory Risk Indicator for Necrotizing Fasciitis (LRINEC) score. An LRINEC >6 has been found to have a positive predictive value of 92% and an LRINEC <6 has a negative predictive value of 96% [7]. Early diagnosis is important, with delay in surgical debridement being associated with increased mortality [8].

There are multiple risk factors that may contribute to a higher incidence of NF, including chronic diseases such as diabetes mellitus (DM), which can be present in as many as 60% of documented NF cases [9]. Diabetes has proven to be an independent risk factor in the development of chronic kidney disease (CKD) and end-stage renal disease (ESRD) [10]. Due to elevation in these laboratory markers, this can pre-dispose patients with DM to having a higher baseline LRINEC score, making the diagnosis difficult in this subset of patients.

As previously mentioned, diabetes is the most common comorbidity present in NF patients [9]. There is a paucity of literature comparing NF patients with and without diabetes. The goal of this study is to further evaluate the differences between patients with diabetes and those without who have operatively confirmed NF, in order to better understand clinical characteristics and trends that can assist clinicians in expediently diagnosing and treating NF.

## Materials and methods

Patient identification and data collection

After formal Institutional Review Board approval (IRB Number: 1751197), all patients diagnosed with NF from July 2015 to January 2021 at three hospitals within a single hospital system were retrospectively reviewed. The inclusion criteria included (1) patients who went to the operating room (OR) for pre-operative concern of NF, (2) patients who had a diagnosis of NF confirmed in the operating room, (3) patients with pre-operative laboratory values available to calculate the LRINEC score (Table 1), and (4) patients aged ≥ 18 years. The exclusion criteria included (1) patients who did not go to the OR or did not have a final diagnosis of NF, (2) patients who had NF of an area other than an extremity, and (3) patients without documentation available detailing laboratory values. The electronic medical record was used to obtain demographic data including age, gender, body mass index (BMI), presence of DM, laboratory values, presence of soft tissue free air on computed tomography (CT) scans, and surgery(s) performed. The LRINEC score was calculated for each patient using the previously described method [7]. The glycemic gap was calculated in the diabetes group only and was determined by the equation as described by Chen et al. [11].

**Table 1:** Laboratory Risk Indicator for Necrotizing Fasciitis Score

Variable	Score
C-reactive Protein, mg/L	
< 150	0
> 150	4
White Blood Cell Count, per mm^3^	
< 15	0
15-25	1
> 25	2
Hemoglobin, g/dL	
> 13.5	0
11-13.5	1
< 11	2
Sodium, mmol/L	
> 135	0
< 135	2
Creatinine, mg/dL	
< 1.41	0
> 1.41	2
Glucose, mg/dL	
< 100	0
> 100	1

Analysis of CT scans was performed by two junior authors. All available cuts were analyzed and evaluated for the presence or absence of free air in the fascial planes. A positive finding was reported as the presence of air in the soft tissue abutting or deep to the fascial plane. Operative reports were analyzed, and NF was confirmed if there was evidence of necrotic deep fascia or the presence of intra-operative dishwasher fluid that tracked along the facial planes. Culture results and the need for repeat operations were also reviewed.

Statistical analysis

After data collection, statistics were analyzed with means, ranges, and confidence intervals calculated for continuous variables and compared using Student’s t-tests. Categorical variables were reported as percentages and analyzed using the chi-squared test. A Shapiro-Wilk test was run on all continuous variables in the study to determine if variables were normal (parametric) or non-normal (non-parametric). After this test was completed, the type of statistical test was determined for these variables. Parametric variables were reported via independent t-tests. Non-parametric variables were reported with the use of a Mann-Whitney U test. A p-value of 0.05 was considered signiﬁcant. Microsoft Excel (Redmond, WA) was used for data computation.

## Results

From 2015 to 2021, a total of 115 patients underwent surgical intervention due to concern for NF of an extremity. Twenty-three patients were lacking preoperative laboratory values and were excluded, leaving 92 patients in the analysis. Table 2 demonstrates the demographic data for the two groups. There were 55 patients in the diabetes group and 37 in the non-diabetes group. All patients in the diabetes group had type 2 DM. Patients with controlled DM were considered to have a hemoglobin A1c (HbA1c) of less than 7.0. There was a significant difference in age between the groups with the non-diabetes group being younger. Additionally, the diabetes group had a higher average BMI and significantly more patients with CKD, while the non-diabetes group had significantly more smokers. The average HbA1c for the diabetes group was 9.2 versus 5.3 in the non-diabetes group. The calculated glycemic gap was 217.5 in the diabetes group.

**Table 2:** Demographic Data Noncategorical variables are given as mean values ± standard deviations. Categorical variables are given as absolute numbers with percentages in parentheses. CKD: chronic kidney disease

Variable	Diabetes	Non-diabetes	P-Value
Patients (Number)	55	37	-
Age (Years)	57.7 ± 12.3	44.1 ± 12.5	< 0.0001
Sex (Male)	36 (65.5%)	25 (67.6%)	0.833
Body Mass Index (kg/m^2^)	34.7 ± 8.4	28.8 ± 9.5	0.039
Smoker (Yes)	25 (45.5%)	27 (73.0%)	0.0090
CKD (Yes)	37 (67.3%)	2 (5.4%)	0.0026
Penetrating Trauma (Yes)	8 (14.5%)	6 (16.2%)	0.855

The clinical data is represented in Table 3. Patients with diabetes had a significantly higher C-reactive protein (CRP), creatinine, and blood glucose, while there was no difference found between the white blood cell count (WBC), hemoglobin (Hb), or sodium. The average LRINEC score for the non-diabetes cohort with confirmed NF was found to be 7.24 compared to 9.02 in the diabetes cohort (p = 0.027). Additionally, CT demonstrated free air in 58.2% of patients in the diabetes group, but only 37.8% of the non-diabetes group (p = 0.07).

**Table 3:** Clinical Data Noncategorical variables are given as mean values ± standard deviations. LRINEC Score: Laboratory Risk Indicator for Necrotizing Fasciitis Score

Laboratory Values	Diabetes	Non-diabetes	P-Value
C-Reactive Protein (mg/L)	277.48 ± 105.83	222.37 ± 126.88	0.026
White Blood Cell Count (x10K/uL)	18.83 ± 7.81	19.21 ± 9.76	0.897
Hemoglobin (g/dL)	10.14 ± 1.97	10.94 ± 3.03	0.234
Sodium (mmol/L)	132.58 ± 5.56	132.62 ± 6.08	0.974
Creatinine (mg/dL)	2.36 ± 2.10	1.19 ± 0.79	0.002
Glucose (mg/dL)	273.60 ± 171.16	129.62 ± 47.57	<0.001
LRINEC Score	9.02 ± 2.09	7.24 ± 2.84	0.027

The operative variables are presented in Table 4. The diabetes cohort was significantly more likely to undergo amputation at the time of their initial surgery (p < 0.0001). The most common amputation level in the diabetes group was a below-knee amputation (50.0%), while an above-knee amputation (50.0%) was the most likely amputation level in the non-diabetes group. Patients with clinical evidence of rapid progression and imaging findings with free air underwent initial amputation and were presumed to have NF. Each group had a similar incidence of repeat surgery, around 70%. In the diabetes group, there were 58 repeat surgeries for an average of 1.5 surgeries per person. For the non-diabetes group, there were 35 repeat surgeries for an average of 1.3.

**Table 4:** Operative Variables Categorical variables are given as absolute numbers with percentages in parentheses. BKA: Below Knee Amputation AKA: Above Knee Amputation

Laboratory Values	Diabetes	Non-diabetes	P-Value
Intraoperative Dishwater Fluid (Yes)	30 (54.5%)	13 (35.1%)	0.067
Grey Necrotic Deep Fascia (Yes)	18 (32.7%)	10 (27.0%)	0.617
Amputation (Yes)	38 (69.1%)	8 (21.6%)	<0.0001
Level of Amputation	Fingers: 1 (2.6%)	Fingers: 0 (0%)	-
	Transforearm: 3 (7.8%)	Transforearm: 0 (0%)	
	Transhumeral: 1 (2.6%)	Transhumeral: 0 (0%)	
	Toes/Foot: 2 (5.3%)	Toes/Foot: 0 (0%)	
	BKA: 19 (50.0%)	BKA: 2 (25.0%)	
	Through knee: 1 (2.6%)	Through knee: 1 (12.5%)	
	AKA: 11 (28.9%)	AKA: 4 (50.0%)	
	Hip Disarticulation: 0 (0%)	Hip Disarticulation: 1 (12.5%)	
Repeat Surgery	39 (70.9%)	28 (73.7%)	0.614
Mortality	17 (30.9%)	7 (18.9%)	0.199

There were 12 patients with controlled DM with an average HbA1c of 6.28 compared to 10.1 for the uncontrolled group. The average LRINEC score in the controlled DM group was 9.42 compared to 8.91 in the uncontrolled group. Patients with controlled DM underwent amputation at primary procedure 83.3% of the time compared to 65.1%. The in-hospital mortality rate for the uncontrolled DM group was 41.6% compared to 27.9% in the uncontrolled group.

Culture results are presented in Table 5. A culture was considered polymicrobial if more than one organism was isolated on culture results. Culture results for the diabetes group showed that polymicrobial cultures (38.2%) were the most common result, followed by Methicillin-resistant Staphylococcus aureus (MRSA) (29.1%). There were nine patients that had no growth on cultures (16.4%). In contrast, in the non-diabetes group, MRSA was the most common isolated organism (35.1%), followed by polymicrobial (27.0%). Furthermore, 13.5% of patients had no growth in cultures. The in-hospital mortality rate for diabetes and non-diabetes cohorts were 30.9% and 18.9%, respectively (p = 0.199).

**Table 5:** Culture Results AS: Actinomyces species BF: Bacteroides fragilis C: Clostridium species CA: Cutibacterium acnes CaK: Candida krusei CB: Corynebacterium CF: Citrobacter freundii CK: Citrobacter koseri CNS: coagulase negative Staphylococcus D: Diptheroids EbC: Enterobacter cloacae EiC: Eikenella corrodens EC: Escherichia coli EF: Enterococcus faecalis F: Fusibacterium KP: Klebsiella pneumonia ML: Micrococcus luteus MM: Morganella morganii MRSA: Methicillin-sensitive Staphylococcus aureus MSSA: Methicillin-sensitive Staphylococcus aureus PA: Pseudomonas aeruginosa PH: Proteus hauseri PM: Proteus mirabilis PrM: Prevotella melaninogenica PO: Prevotella oralis PS: Paeniclostridium sorellii SA: Streptococcus agalactiae StA: Streptococcus anginosus StM: Stenotrophomonas maltophilia SD: Streptococcus dysgalactiae SE: Staphylococcus epidermidis SM: Serratia marcescens SP: Streptococcus pyogenes SS: Staphylococcus simulans SV: Streptococci viridians

Laboratory Values	Diabetes	Non-diabetes
Positive Culture Result	46 (83.6%)	32 (86.5%)
MRSA	16 (29.1%)	13 (35.1%)
Polymicrobial	21 (38.2%)	10 (27.0%)
Culture Results (Monomicrobial)	MRSA: 16	MRSA: 13
	SA: 3	SA: 3
	EbC: 1	SP: 2
	EF: 1	C: 1
	KP: 1	CA: 1
	ML: 1	EbC: 1
	MSSA: 1	SP: 1
	SV: 1	
Culture Results (Polymicrobial)	MRSA, SA: 2	BF, MM, SD: 1
	AS, StA, SV: 1	CaK, SE: 1
	BF, EC: 1	D, PS: 1
	BF, EF, PrM: 1	EiC, PM, PO, SV: 1
	BF, PH: 1	EC, SV:1
	BF, StA, SV, SD: 1	MSSA, SV: 1
	CF, SM: 1	PA, SE: 1
	CK, EF, PA: 1	PM, SV: 1
	CNS, SV: 1	StM, SS: 1
	EbC, MRSA: 1	SE, SV: 1
	EC, MRSA, SA: 1	
	EF, SM: 1	
	F, MSSA: 1	
	KP, SA, SV: 1	
	MRSA, PM: 1	
	MSSA, SA: 1	
	PA, SP: 1	
	StA, SV: 1	
	SA, SP: 1	
	SA, SV: 1	

## Discussion

NF often leads to life-threatening metabolic changes and requires emergent operative management to aid in treatment. The LRINEC score was designed to act as a tool to assist in the quick diagnosis of NF [7]. Tan et al. reported that patients with diabetes have a significantly elevated LRINEC score compared to their counterparts and demonstrated that a cutoff of 8 or greater was significantly more sensitive in patients with diabetes compared to patients without diabetes [12]. Along the same lines, it has been reported that in patients with DM, an LRINEC score < 7 has a strong negative predictive value [13]. In the present study, it was found that patients with DM had a significantly higher LRINEC score than their counterparts (p = 0.027). Additionally, in our study, there was a high sensitivity (92.7%) and specificity (37.8%) for NF in patients with DM with an LRINEC score > 7. Like the previous studies, we found a low positive predictive value of 68.9% when using a score of > 7 [13]. This underscores the difficulty in diagnosing NF in patients with DM, given their baseline elevations in laboratory markers.

Findings related to NF on CT scans can be variable. The most common finding reported is fascial thickening found in up to 80% of patients [14]. It has been reported that the presence of air tracking along fascial planes is only present in about 55% of patients [14]. In the present study, a CT scan demonstrated free air in 58.2% of patients in the diabetes group, compared to only 37.8% of patients in the non-diabetes group (p = 0.071). Due to the air only being present in a little over half of the patients with NF and less than half of the non-diabetes group, a high clinical suspicion remains necessary, and a CT scan should not be used to rule NF out.

The present study demonstrated polymicrobial infections as the most common cultured organism encountered in the diabetes cohort (38.2%), followed by MRSA (29.1%). In the non-diabetes group, MRSA was the most isolated organism (35.1%), followed by polymicrobial (27.0%). Khamuan et al. reported in their series that *Streptococcus pyogenes* was the most common causative organism in both patients with diabetes and patients without diabetes [15]. Similar to our findings, Chen et al. as well as others have reported that polymicrobial infections represent the most common agent in the DM population (26.2%) [11]. This said it can often be difficult to confirm the causative organism. Yu et al. showed that the causative organism could be identified in only 81.9% of cases [16]. Along the same lines, a causative organism was isolated in 55.9% of wound cultures and in 48% of blood cultures in a single-center study of patients with NF [17]. The current series showed that organisms were identified in 83.6% of patients with diabetes and in 86.5% of patients without. Overall, 15.2% of patients did not have any organism isolated. Due to the many causative organisms and the difficulty of isolating a specific organism, the initial broad-spectrum antibiotic is recommended until culture results can be determined.

The rapidly progressive nature of NF can often lead to amputation due to the need for infection control. Rates demonstrate that this can occur in up to 29.2% of patients [18]. Additionally, it has been shown that patients with DM have a significantly higher chance of amputation [18,19]. Another similar study reports DM to be an independent risk factor for amputation [20]. The current study found patients with DM had a significantly higher rate of amputation when diagnosed with NF (p < 0.0001) with nearly 70% of these patients requiring amputation. Interestingly, patients with well-controlled DM had a higher rate of amputation (83.3%) compared to patients with uncontrolled DM (65.1%).

The overall in-hospital mortality rate for the current study was 26.1% for patients both with and without DM, which is similar to published rates. Espander et al. found an overall mortality rate of 20.8% in their series [18]. Cheng et al. reported an overall mortality rate of 29.7% with no difference in rates between patients with diabetes and those without [20]. Overall, NF is a very serious disease that carries a significant risk of mortality, underscoring the need for prompt recognition and treatment to help reduce this risk.

This study has several limitations. As a retrospective review, our analysis contains inherent bias associated with retrospective studies. Along the same lines, we had a relatively small patient cohort; however, all had been taken to the OR and a diagnosis of NF was confirmed, which we believe strengthens our conclusions. Further, NF is a relatively rare disease and can make large cohorts difficult to obtain. Future areas of study include comparisons of the LRINEC score in patients with diabetes with NF to patients with diabetes without NF and those with NF to those with other non-necrotizing soft tissue infections including cellulitis and abscess to further isolate the best predictive algorithm.

## Conclusions

This study demonstrated that patients with diabetes with confirmed NF of an extremity had a statistically significant higher LRINEC score compared to their counterparts. Given this finding, the use of LRINEC scores for diagnosis should be considered in combination with the patient’s diabetes status, with the understanding that baseline scores may be higher in patients diagnosed with diabetes. Along these lines, a higher index of suspicion may be necessary for patients without DM. Additionally, patients with DM were significantly more likely to undergo amputation at the primary procedure than patients without DM. In the non-diabetes group, MRSA followed by polymicrobial infections were the most common organisms isolated, while the inverse was true for the diabetes group. The overall mortality rate of NF was 26.1%.
